# Supplementary material for: Co-developing a health promotion programme for indigenous youths in Brazil: A concept mapping report
Source: PLoS One. 2023 Feb 15;18(2):e0269653. doi: 10.1371/journal.pone.0269653 (PMC9931109; doi:10.1371/journal.pone.0269653)
Supplement: S1 Table — (DOCX) [file pone.0269653.s001.docx]

**Supplementary table 1**. Statements generated from prompt 1: To be happy… and prompt 2: To have a healthy body… grouped in clusters and the average rate for importance and feasibility

|  | **Themes**  **(B = average bridging score)** | **# of items** | **Examples of items** | **Average Importance Score*** | **Average Feasibility Score*** |
| --- | --- | --- | --- | --- | --- |
| **Students’ perceptions on happiness components** | **Family**  (B = 0.05) | 16 | 1. To have family union; 6. Financial independence; 16. Receive family care. ; 18. Improve relationship with my mom; 20. Defend family in disputes and discussions; 23. To have a good job; 27. I need paternal presence; 29. My family feel happier; 33. Have a baby brother; 40. My father needs to get a job; 42. Make my family happy; 44. Being with my father and mother; 46. To have peace; 51. Celebrate my birthday; 67. My father needs to stop drinking; 68. My parents need stay together as a couple. | 2.65 | 2.70 |
|  | **Socio-economic circumstances**  (B = 0.24) | 10 | 19. Building a family; 22. Own a house; 30. To have joy; 32. Eat tasty foods; 34. Have access to mobile phone; 45. My Family; 49. To have compassion; 56. Doing my homework with my mother; 59. Have money; 66. Feed me well. | 2.63 | 2.73 |
|  | **Respect**  (B = 0.30) | 14 | 7. Respect among people; 24.To have faith; 37. To buy toys; 39. My parents to stop fighting; 50. Visit my relatives; 55. My mom moving house; 63. Not being alone; 64. My aunt to be home; 65. Don't get beaten by my mother; 69. Do not be cold; 70. My relatives not to die; 71. Traveling; 72. My mum gifting me soccer shoes; 73. Have love. | 2.42 | 2.48 |
|  | **Sport**  (B = 0.60) | 10 | 8. Invest money in sport; 9. Mortality decrease; 10. To be a professional soccer player; 15. Have physical and mental health; 25. Having a boy or girlfriend; 26. Play sports; 36. Not to be bullied; 53. To dance “funk”; 61. Not being with strangers; 62. Do not be grounded. | 2.31 | 2.41 |
|  | **School**  (B = 0.07) | 15 | 2. 15 minutes of break at school; 3. More fun classes; 4. Practical classes; 11. To be asked less question in class; 13. Physical education class on Monday; 21. To get good grades; 31. To have friends; 35. To be happy at school; 38. My friends to stop fighting; 41. See my teacher happy; 47. To paint; 48. To draw; 54. A handsome/good teacher; 57. To read; 60. Play at school. | 2.40 | 2.67 |
|  | **Education**  (B = 0.14) | 8 | 5. Be intelligent or smart; 12. Have a library at school; 14. Leisure activities; 17. To graduate from high school; 28. To study; 43. Being with my teacher; 52. Play with my teacher; 58. Know how to read. | 2.53 | 2.74 |
|  | **TOTAL** | 73 |  | 2.49 | 2.62 |
| **Students’ perception on healthy body** | **Diet**  (B = 0.06) | 11 | 1. Eat fruits; 2. Eat vegetables; 3. Eat protein; 4. Eat less fat; 5. Healthy eating; 7. Include fruits and vegetables in the school snack; 8. Healthy snack to be provided at school; 12. Drink water; 25. Eat natural food; 27. Eat a variety of food. 32. Abundance of food. | 2.60 | 2.61 |
|  | **Physical activity**  (B = 0.14) | 10 | 6. Sleeping well; 9. Sport at school; 10. Exercise class; 11. Practice daily exercise; 13. Respect for the body; 16. Friendly play; 18. Have cold water in the drinking fountain; 22. Practical/applied classes; 24. Playing sports; 33. Physical activity. | 2.68 | 2.75 |
|  | **Local environment**  (B = 0.54) | 7 | 14. Doing what I want with my body; 19. More space for trash bins; 20. Trash bin for recycling; 21. Add new creative courses; 30. Cultivation/Planting/ growing my own food; 34. Contemplation of nature/ respect of nature; 35. Access to natural resources. | 2.58 | 2.50 |
|  | **Well being**  (B = 0.32) | 9 | 15. Have joy and happiness; 17. Behave well; 23. Have a library; 26. Having enough money; 28. Receiving family care; 29. Strong family relationship; 31. Have a home/place to live; 36. Strong community; 37. Have more recreational activities | 2.64 | 2.58 |
|  | **TOTAL** | 37 |  | 2.63 | 2.61 |

*Score out of 3.
