# Supplementary material for: Co-developing a health promotion programme for indigenous youths in Brazil: A concept mapping report
Source: PLoS One. 2023 Feb 15;18(2):e0269653. doi: 10.1371/journal.pone.0269653 (PMC9931109; doi:10.1371/journal.pone.0269653)
Supplement: S2 Table — (DOCX) [file pone.0269653.s002.docx]

**Supplementary table 2** Adult stakeholders’ statements grouped in clusters and the average rate for importance and feasibility.

|  | **Themes**  **(B = average bridging score)** | **# of items** | **Statements** | **Average Importance Score*** | **Average Feasibility Score*** |
| --- | --- | --- | --- | --- | --- |
| **Improving the health of young people in the village and school** | **Relationships**  (B = 0.10) | 6 | 1. Improve relationship with family; 29. Analyse most common problems; 30. Use interesting/ creative strategies; 45. Work as a team; 47. Provide adequate space; 50. Include the actions as a school schedule. | 4.56 | 4.07 |
|  | **Health issues**  (B = 0.20) | 5 | 8. Discuss the importance of disease prevention; 12. Work with a multidisciplinary team; 32. Discuss about sexuality; 33. Discuss about addiction; 34. Discuss about alcoholism. | 4.57 | 4.13 |
|  | **Prevention at Polo Base**  (B = 0.33) | 7 | 7. To perform preventive actions (“day of prevention”); 21. Joint work between school and health team; 28. Provide information about users' rights of the health service; 37. Provide training on the topics covered; 44. Address topics such as: health care items/priorities; 46. Equity in service; 49. Discuss about mental health. | 4.60 | 4.04 |
|  | **Access to health care**  (B = 0.46) | 4 | 22. Train AIS; 23. Engage people to look for the prevention health unit; 27. Improve the "queue" for attending and scheduling exams; 39. Include all health team. | 4.52 | 3.96 |
|  | **Communication with young people**  (B = 0.13) | 11 | 4. Improve relationship with young people; 5. Work on alcoholism problem; 9. Provide knowledge about disease to the young people; 10. Follow up on the daily of young people; 11. Monitor behaviour changes; 13. Listen to the young people; 14. Clarify the doubts of young people; 15. Search among young people on which topics they are interested in; 16. Use the language of young people; 24. Improve dialogue between parents and children; 25. Provide care/treatment when necessary. | 4.41 | 3.77 |
|  | **Community life**  (B = 0.68) | 6 | 2. Rescue the culture; 17. Search for the most prevalent conditions among them and develop specific actions; 18. Be integrated in the life of the community; 31. Discuss about nutrition; 35. Encourage the sport; 36. Encourage leisure activities. | 4.61 | 4.12 |
|  | **Raising awareness**  (B = 0.30) | 5 | 3. Know the family eating behaviour; 6. Use active methodologies, multimedia, group activities, theatre, jokes. 19. Give awareness lectures; 43. Realize when something is wrong with their health; 51. Close relationships. | 4.37 | 3.74 |
|  | **School support**  (B = 0.50) | 7 | 20. Include the community in the activity; 26. Provide appropriate support; 38. Commitment of professionals to carrying out the activities; 40. Plan and organize activities; 41. Train teachers; 42. Students as active actors in the activities; 48. Provide material. | 4.62 | 4.15 |
|  | **TOTAL** | 51 |  | 4.53 | 4.00 |
| **Embedding Indigenous Health Agent in school** | **Method**  (B = 0.35) | 9 | 1. Working together with teachers; 2. Work towards learning (pedagogical approach); 3. Use interesting methodologies; 5. Raising awareness among leaders and teachers of the "Health in School" importance; 6. Encourage the exchange of information; 7. Improve the relationship between the health team and teachers; 8. Create partnerships between the education and health departments; 12. Empower the teacher to work on health issues; 14. Develop activities geared toward students. | 4.48 | 3.94 |
|  | **Indigenous community health**  (B = 0.75) | 8 | 4. Train the AIS to work with children; 9. Make AIS activities continuous in school; 10. Stimulate the desire in the AIS; 11. Provide opportunity to work together; 13. Buy the resources needed to develop the action; 15. Reschedule working hours of AIS; 16. Reschedule the work routine of AIS; 17. The school is responsible for ensuring the participation of students. | 4.22 | 3.69 |
|  | **TOTAL** | 17 |  | 4.35 | 3.82 |

*Score out of 5.
