# Supplementary figures and images for: Co-developing a health promotion programme for indigenous youths in Brazil: A concept mapping report
Source: PLoS One. 2023 Feb 15;18(2):e0269653. doi: 10.1371/journal.pone.0269653 (PMC9931109; doi:10.1371/journal.pone.0269653)

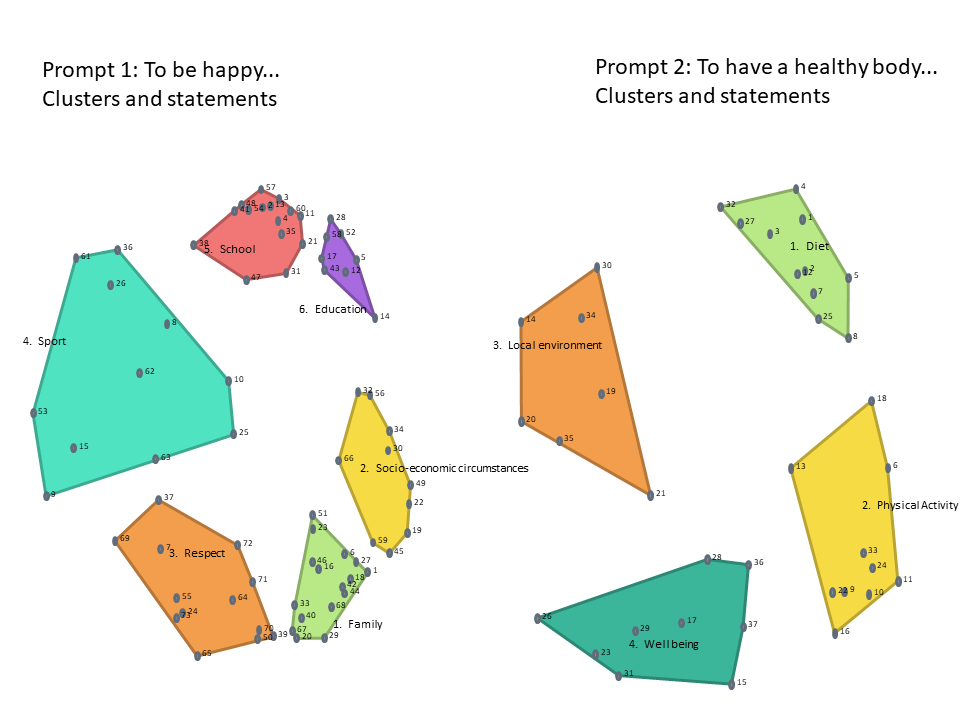

Supplement: S1 Fig — Ideas that were grouped together more often appear closer to each other on the map. Ideas never/rarely grouped together appear widely separated on the map. Clusters are groups of ideas that were grouped together most often and reflect ideas that are conceptually related according to the participants in this group. The defined cluster names in this concept maps are: 1- Family, 2- Socioeconomic circumstances, 3- Respect, 4- Sports, 5- School, 6- Education. Youths’ cluster map for prompt 2, ‘To have a healthy body it is necessary to…’ In this map, each point reflects one idea. Ideas that were grouped together more often appear closer to each other on the map. Ideas never/rarely grouped together appear widely separated on the map. Clusters are groups of ideas that were grouped together most often and reflect ideas that are conceptually related according to the participants in this group. The defined cluster names in this concept maps are: 1- Nutrition pattern, 2- Physical activity, 3- Well-being, 4- Local environment. (TIF) [file pone.0269653.s006.tif]

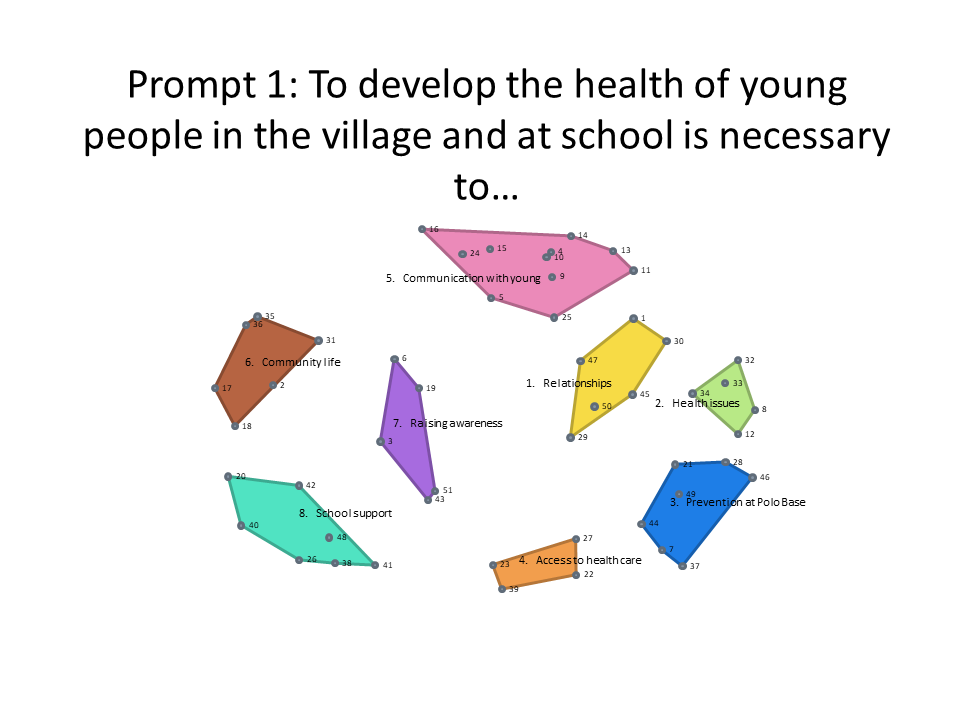

Supplement: S2 Fig — Ideas that were grouped together more often appear closer to each other on the map. Ideas never/rarely grouped together appear widely separated on the map. Clusters are groups of ideas that were grouped together most often and reflect ideas that are conceptually related according to the participants in this group. The defined cluster names in this concept maps are: 1- Relationships, 2- Communication with young people, 3- Health issues, 4- Raising awareness, 5- Prevention at Polo Base, 6- Access to healthcare, 7- School support, 8- Community life. (TIF) [file pone.0269653.s007.tif]
