# Supplementary material for: Co-developing a health promotion programme for indigenous youths in Brazil: A concept mapping report
Source: PLoS One. 2023 Feb 15;18(2):e0269653. doi: 10.1371/journal.pone.0269653 (PMC9931109; doi:10.1371/journal.pone.0269653)
Supplement: S1 Dataset — (DOCX) [file pone.0269653.s008.docx]

**Point maps - Youth**

**Point map – Adults**

**Point rating map for importance and feasibility– Youths**

**Adults**

**Table 1.** Themes, bridging value, statements, drawings and average importance and feasibility ratings for adolescents.

|  | **Themes**  **(B = average bridging score)** | **# of items** | **Statements*** | **Drawing example for the theme**** | **Average Importance Rating*** | **Average Feasibility Rating*** |
| --- | --- | --- | --- | --- | --- | --- |
| **Prompt 1: “To be happy it is necessary to…” (SV=0.25)** | **Family**  (B = 0.05) | 16 | **1. Family union**; 6. Financial independence; **16. Family care;** **18. Improve relationship with mom**; **20. Defend family in disputes;** **23. Have a good job**; 27. Paternal presence; **29. Happiness in my family**; 33. Have a baby brother; **40. My father to get a job;** **42. Make my family happy; 44. Being with my parents; 46. Peace;** 51. Celebrate my birthday; 67. My father to stop drinking; 68. My parents not divorcing. | 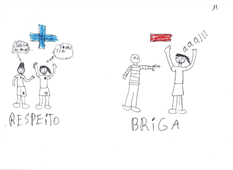 | 2.65 | 2.70 |
|  | **Socio-economic circumstances**  (B = 0.24) | 10 | **19. Building a family; 22. Own a house; 30. Joy**; **32. Eat tasty foods**; 34. Have access to mobile phone; **45. My family;** **49. Compassion**; 56. Doing my homework with my mother; **59. Have money; 66. Feed me well.** | 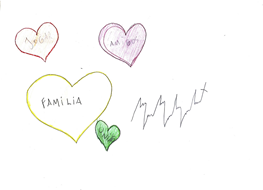 | 2.63 | 2.73 |
|  | **Respect**  (B = 0.30) | 14 | **7. Respect among people; 24. Faith**; 37. To buy toys; 39. My parents to stop fighting; 50. Visit my relatives; 55. My mom moving out; 63. Not being alone; 64. My aunt to be home; 65. Don't get beaten by my mother; 69. Not be cold; 70. My relatives not dying; **71. Traveling**; 72. My mum gifting me soccer shoes; 73. Love. | 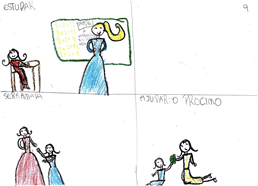 | 2.42 | 2.48 |
|  | **Sport**  (B = 0.60) | 10 | **8. Investment in sport;** 9. Mortality decrease; 10. Be a professional soccer player; 15. Have physical and mental health; 25. Having a boy or girlfriend; **26. Play sports;** 36. Not to be bullied; 53. To dance “funk”; 61. Not being with strangers; 62. Not be grounded. | 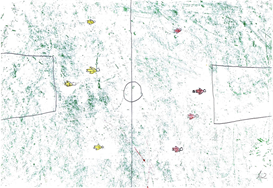 | 2.31 | 2.41 |
|  | **School**  (B = 0.07) | 15 | 2. 15 minutes of break at school; **3. More fun classes**; 4. Practical classes; 11. Less question in class; **13.** **Physical education class**; **21. Get good grades;** **31. Have friends; 35. Be happy at school**; 38. My friends to stop fighting; **41. See my teacher happy;** 47. To paint; 48. To draw; 54; A handsome/good teacher **57. Read;** 60. Play at school. | 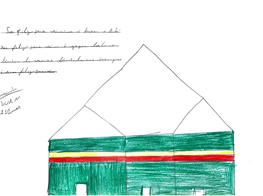 | 2.40 | 2.67 |
|  | **Education**  (B = 0.14) | 8 | 5**. Be intelligent;** 12. Have a library at school; 14. Leisure activities; 17. Graduate from high school; **28. To study;** 43. Being with my teacher; 52. Play with my teacher; **58. Know how to read.** | 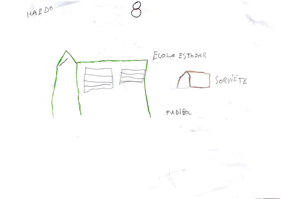 | 2.53 | 2.74 |
|  | **TOTAL** | 73 |  |  | 2.49 | 2.62 |
| **“To have a healthy body it is necessary to…” (SV=0.27)** | **Diet**  (B = 0.06) | 11 | **1. Eat fruits;** 2. Eat vegetables; 3. Eat protein; 4. Eat less fat; **5. Healthy eating;** 7. Include fruits and vegetables in the school snack; 8. Healthy snack to be provided at school; **12. Drink water;** 25. Eat natural food; 27. Eat a variety of food. 32. Abundance of food. | 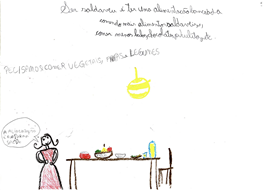 | 2.60 | 2.61 |
|  | **Physical activity**  (B = 0.14) | 10 | **6. Sleeping well; 9. Sport at school**; 10. Exercise class; 11. Practice daily exercise; **13. Respect for the body; 16. Friendly play;** 18. Have cold water in the drinking fountain; **22. Practical/applied classes**; **24. Playing sports; 33. Physical activity.** | 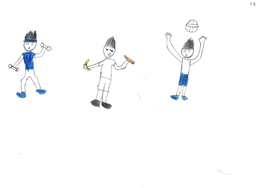 | 2.68 | 2.75 |
|  | **Local environment**  (B = 0.54) | 7 | 14. Doing what I want with my body; 19. More space for trash bins; 20. Trash bin for recycling; 21. Add new creative courses; 30. Cultivation/Planting/ growing my own food; 34. Contemplation of nature/ respect of nature; 35. Access to natural resources. | 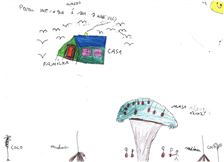 | 2.58 | 2.50 |
|  | **Well being**  (B = 0.32) | 9 | **15. Have joy and happiness;** **17. Behave well;** 23. Have a library; 26. Have enough money; **28. Receive family care**; **29. Strong family relationship**; 31. Have a home/ place to live; **36. Strong community;** 37. More recreational activities | 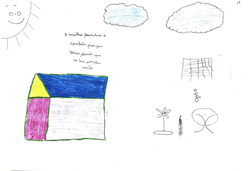 | 2.64 | 2.58 |
|  | **TOTAL** | 37 |  |  | 2.63 | 2.61 |

*Score out of 3,

** Statements in bold refer to items in the upper right quadrant of Go-Zone (high importance and feasibility rating)

**Table 2**. Themes, bridging value, statements and average importance and feasibility rating for adults

|  | **Themes**  **(B = average bridging score)** | **# of items** | **Statements**** | **Average Importance Rating*** | **Average Feasibility Rating*** |
| --- | --- | --- | --- | --- | --- |
| ***“To develop the health of young people in the village and at school is necessary to…”* (SV=0.33)** | **Relationships**  (B = 0.10) | 6 | **1. Improve family relationships**; 29. Analyse most common problems; 30. Use interesting/creative strategies; **45. Work as a team;** 47. Provide adequate space; 50. Include the actions in school schedule. | 4.56 | 4.07 |
|  | **Health issues**  (B = 0.20) | 5 | **8. Discuss the importance of disease prevention**; 12. Work with a multidisciplinary team; **32.** **Discuss about sexuality; 33. Discuss about addiction; 34. Discuss about alcoholism.** | 4.57 | 4.13 |
|  | **Prevention at Polo Base**  (B = 0.33) | 7 | **7. Conduct prevention events (e.g. day of prevention); 21. Joint work between school and health team;** 28. Provide information about users' rights of the health service; **37. Provide training on the topics covered; 44. Address health care items/priorities**; 46. Equity in service; 49. Discuss about mental health. | 4.60 | 4.04 |
|  | **Access to health care**  (B = 0.46) | 4 | 22. Train AIS; **23. Engage people to look for the prevention health unit**; 27. Improve the "queue" for attending and scheduling exams; **39. Include all health team.** | 4.52 | 3.96 |
|  | **Communication with young people**  (B = 0.13) | 11 | 4. Improve relationship with young people; 5. Work on alcoholism problem; 9. Provide disease knowledge; 10. Follow up on the daily life of young people; 11. Monitor behaviour changes; 13. Listen to the young people; 14. Clarify the doubts of young people; 15. Explore which topics are young people interested in; 16. Use the language of young people; **24. Improve dialogue between parents and children;** 25. Provide care/treatment when necessary. | 4.41 | 3.77 |
|  | **Community life**  (B = 0.68) | 6 | **2. Rescue the culture;** 17. Search for the most prevalent conditions among them and develop specific actions; **18. Be integrated in the life of the community;** 31. Discuss about nutrition; **35. Encourage the sport;** **36. Encourage leisure activities.** | 4.61 | 4.12 |
|  | **Raising awareness**  (B = 0.30) | 5 | 3. Know the family eating behaviour; 6. Use interactive methodologies; 19. Give awareness lectures; **43. Raise health awareness**; 51. Close relationships. | 4.37 | 3.74 |
|  | **School support**  (B = 0.50) | 7 | **20. Include the community in the activity;** 26. Provide appropriate support; **38. Commitment of professionals to carrying out the activities**; 40. Plan and organise activities; **41. Train teachers;** 42. Students as active actors in the activities; 48. Provide material. | 4.62 | 4.15 |
|  | **TOTAL** | 51 |  | 4.53 | 4.00 |
| **Embedding Indigenous Health Agent in school (SV=0.23)** | **Method**  (B = 0.35) | 9 | **1. Work with teachers;** **2. Work towards learning (pedagogical approach)**; **3. Use interesting methodologies; 5. Raise awareness among leaders and teachers on the "Health in School" importance**; 6. Encourage the exchange of information; 7. Improve the relationship between the health team and teachers; 8. Create partnerships between the education and health departments; **12. Empower teachers to work on health issues;** **14. Develop activities geared toward students.** | 4.48 | 3.94 |
|  | **Indigenous community health**  (B = 0.75) | 8 | **4. Train the AIS to work with children;** 9. Make AIS activities continuous in school; 10. Motivate the AIS; **11. Provide opportunity to work together;** 13. Procure the required equipment; 15. Reschedule working hours of AIS; 16. Reschedule the work routine of AIS; 17. School’s responsibility to ensure students’ participation. | 4.22 | 3.69 |
|  | **TOTAL** | 17 |  | 4.35 | 3.82 |

*Score out of 5.

** Statements in bold refer to items in the upper right quadrant of Go-Zone map (high importance and feasibility rating)
