# Supplementary material for: Co-developing a health promotion programme for indigenous youths in Brazil: A concept mapping report
Source: PLoS One. 2023 Feb 15;18(2):e0269653. doi: 10.1371/journal.pone.0269653 (PMC9931109; doi:10.1371/journal.pone.0269653)
Supplement: Attachment — Submitted filename: Response to reviewers-sh230th June.docx [file pone.0269653.s009.docx]

Dear Editor

**Re:** **Readiness for embedding places of worship into the primary care pathway for prevention and control of noncommunicable diseases: a participatory mixed method study in the Caribbean.**

Thank you inviting a re-submission. The reviewers’ comments were very helpful and we believe the paper has been strengthened by their comments.

Below in a point by point response to each comment

We look forward to your further considerations of our paper for inclusion in your journal

| Journal’s requirements | Response |
| --- | --- |
| 1. Please ensure that your manuscript meets PLOS ONE's style requirements, including those for file naming. The PLOS ONE style templates can be found at. | Edited to align with format required. |
| 2. Please provide additional details regarding participant consent. In the ethics statement in the Methods and online submission information,  please ensure that you have specified what type you obtained (for instance, written or verbal, and if verbal, how it was documented and witnessed). | We have amended the methods (p 9)  After consultations meetings about the study (see details below), written informed consent was obtained from all participants and, additionally for students, written parental consent was obtained. The Village Leader gave written consent for the research team to engage with the school and Polo Base. All consents were witnessed by a member of the research team. |
| 3. When reporting the results of qualitative research, we suggest consulting the COREQ guidelines: [http://intqhc.oxfordjournals.org/content/19/6/349](https://eur03.safelinks.protection.outlook.com/?url=http%3A%2F%2Fintqhc.oxfordjournals.org%2Fcontent%2F19%2F6%2F349&data=04%7C01%7Cchristelle.elia%40kcl.ac.uk%7Ce8d147999daf4e26b11908d91b91fd70%7C8370cf1416f34c16b83c724071654356%7C0%7C0%7C637571136096131432%7CUnknown%7CTWFpbGZsb3d8eyJWIjoiMC4wLjAwMDAiLCJQIjoiV2luMzIiLCJBTiI6Ik1haWwiLCJXVCI6Mn0%3D%7C1000&sdata=awvhkmyyo0y0IckNChMrso%2Br1NK4k9yNGQ%2FRTmoObR0%3D&reserved=0). | We have amended to clarify points raised, whilst trying our best to be within the word limit. |
| 4. We note that the grant information you provided in the ‘Funding Information’ and ‘Financial Disclosure’ sections do not match.  When you resubmit, please ensure that you provide the correct grant numbers for the awards you received for your study in the ‘Funding Information’ section | PJCJ, JMD, AJG, ABV, EKF, FAAQ, CP, FCSB, MIMGO and IMAVD were supported by FUNDECT/CONFAP no 05/2018 – MRC: Health Systems Research Networks 2017.Outorga 010/2018.  MOK, CE, PD, IW and SH were supported by Medical Research Council MR/R022739/1  AJG was also supported by the Academy of Medical Science, the Newton Fund NIFR7\1004 |
| 5. Upon re-submitting your revised manuscript, please upload your study’s minimal underlying data set as either Supporting Information files or to a stable, public repository and include the relevant URLs, DOIs, or accession numbers within your revised cover letter | Supplementary file (S6_Data set) added. It includes all anonymised statements, and cluster and point maps which were used to produce the cluster rating maps for importance and feasibility ratings |
| 6. PLOS requires an ORCID iD for the corresponding author in Editorial Manager on papers submitted after December 6th, | 0000-0003-0416-534X |
| 7. We note you have two different versions of Figures 1 and 2 included with your submission, the first embedded in your manuscript (page 10 and page 16) and the second uploaded in the file "Figures (002).docx". So that your figures can be differentiated, can you please update the numbering of the figures and please ensure your figures are uploaded as separate  individual files and not embedded in the manuscript. Further details with regards to figures can be found in the guidelines | Apologies for this error in uploads. All figures were removed from the text and uploaded separately with the correct numbering. |
| 8. We note you have multiple versions of your Tables and Supporting Information files uploaded, to avoid confusion can you please ensure you only have a single version of each included. Ta | Apologies for this error in uploads. We have now included only one version of each document |
| 9. Please include captions for your Supporting Information files at the end of your manuscript, and update any in-text citations to match accordingly. Please see our Supporting Information guidelines for more information: | The captions are now included at the end of the manuscript. |

| Reviewer 1 |  |
| --- | --- |
| 1.More justification up front of why the approach of concept mapping is selected and the researchers’ particular orientation to their work, such as the values and paradigms to which they subscribe, would be helpful. | Thanks you for this important point We have amended the text, including this paragraph on **p10**  Concept mapping methodology uses a participatory approach to combine the tacit knowledge of different groups of stakeholders from different backgrounds. It is also increasingly being used to inform public health interventions and recommended by the World Health Organisation[14,15,16] as a unique method to embed stakeholder perspectives in intervention development [17,18]. Concept mapping enables shared understanding and provides ‘balance of power’ as the method is predominantly participant-led rather than researcher-driven [19,20]. There has been a history of exploitative research practices with Indigenous peoples. This has led Indigenous peoples to regard research led by those outside of their communities with distrust. Concept mapping allowed active participation of the Terena youths and adults across all stages of the study, and thus the prioritisation of Indigenous values, traditions and knowledge in the concept maps. |
| 2.description of the concept-mapping workshops was not as revealing as needed. I was left unclear as to the type of facility they were held in, ….Since the number of youths exceeded methodological recommendations, were they divided into groups beyond asking some to produce artwork? Were they divided in space or time? Were statements by youths written or verbalized? | To clarify, the size of the sample did not exceed recommended sizes. The minimum recommended sample size is 15 and many studies have used more than 30 participants.  We have amended the methods, including the following paragraphs on page 12  Step 1.  For concept mapping to capture the views of those who are responsible for the care of young people, it was necessary to have a broad representation from relevant sectors. Over the decade of working with the Terena, researchers were familiar with key stakeholders in the village, including the Village Leadership, and those working in primary care and education services. On the advice of teachers and the Village Leader, concept mapping was conducted separately with adults and students. At least 15 participants are recommended for concept mapping [19]. All students enrolled in the school, regardless of literacy levels, were eligible. The total number of students in the school were 300, and 40 were invited based on the advice of teachers who took into consideration who might be available on the day and times that concept mapping sessions took place. Students were broadly representative by gender (45% girls, 55% boys) and age (between the ages of 9 to 17 years old). All sessions with the students were led by the same psychologist (CP) and assisted by 2-3 other members of the research team and Indigenous medical students.  The adult group consisted of 15 Indigenous adults, and comprised of 2 community leaders, 5 elementary school teachers, 2 parents of participating students, 2 Indigenous Community Health Workers, 1 nutritionist and 3 nurses. Indigenous adults were eligible for inclusion if they were from the Tereré Village, had an active role in either village leadership, health service delivery, the school or were parents of students enrolled in the school. All sessions with adults were facilitated by 2-3 members of the research team.  Step 2: Indigenous students and adults ‘brainstormed’ statements guided by focused prompts which were previously developed in consultations with Indigenous stakeholders during the approval process. These were: “To be happy it is necessary to…” and “To have a healthy body it is necessary to…” for Indigenous students, and “To develop the health of young people in the village and in school it is necessary that..." for Indigenous adults.  On the advice of teachers, students were subdivided by age as they felt the writing abilities might compromise their contributions. For students aged 9 to 11 years old (n=20), art-enhanced concept mapping was used to encourage their active participation. Students were invited to a classroom and were provided with paper and colouring pencils. The psychologist used a motivational warm-up session asking for examples of the sort of things that made them happy and sad. The first prompt was introduced after it was clear that all students were comfortable with engaging with the topic. They were given the choice of either writing their statements or drawing their response. Students who chose to draw were invited to then write words/sentences that represented their drawings with the aid of a researcher. The same procedure was used with the second prompt. Students created 27 drawings in response to the focused prompts which resulted in 17 different statements.  Older students, aged 12 to 17 years old (n=20), were given pencils and paper and asked to write their statements. |
| 3.Which indigenous stakeholders weighed in on the number of clusters, and I am left concerned about their representativeness | We apologise for the error in the statement ‘The number of clusters were independently chosen by two researchers.‘(page 14) The clusters were decided in consultation with the participants.  We hope the following amendment adds clarity to how the clusters are derived (page 13)  Data were manually entered and analysed using the GroupWisdomTM software (Concept Systems, Inc., Ithaca, NY). In summary, each sorting category was first converted to a 0, 1 co-occurrence matrix, that has as many rows and columns as there are statements. It contains 1 in a cell if the row and column statement pair were placed by the participant in the same category and a 0 if the statements were not sorted together. These matrices were then summed across all participants, yielding a similarity matrix that indicated the number of stakeholders that sorted each pair of statements together. Multidimensional scaling analysis used (dis)similarity data and represented them as distances in Euclidean space. Each statement was assigned an ‘x’ and ‘y’ value from these analyses which were used in hierarchical cluster analysis to partition the statements into non-overlapping clusters. The statements closer to each other on the map are expected to have been sorted similarly across stakeholders. Clusters are groups of ideas that were grouped together most often and reflect the ideas that were conceptually related according to the participants. All scenarios were then discussed with Indigenous participants before choosing a final number of clusters. A bridging value was computed for each statement and the average for each cluster, which ranged from 0 to 1; the lower the value, the better the coherence from sorting across stakeholders. |
| 4. Process whereby the results were interpreted is not fully explained. I do not have a sense of where and how this occurred. I do not know how the youth statements were considered, how the decision to advance a school-Polo Base partnership program was made, how the school infrastructure was taken into consideration, which indigenous participants were consulted, what prompts were used in the discussion, who was present, why the university students were brought in, etc.. | We hope the response above addresses some of these queries  Re ‘the decision to advance a school-Polo Base partnership’ –  We have amended the text, page 9  The idea of schools and the Polo Base working together to promote youth health developed over several years of the research team collaborating with the Terena community. Researchers in our team include nurses and doctors who work with the Polo Base and lecturers who teach Indigenous students. Our participatory concept mapping study sought to explore the viability of the idea of developing a partnership. After consultation on the prompt to be used with adults we decided to keep it broad to see what emerges *‘To develop the health of young people in the village and at school is necessary to…’*  Re Which Indigenous participants were consulted. Please see above for students  We have added to the text, page 9  Adult stakeholders included a broad representation of those who had responsibility for the care of young people: teachers, school director, indigenous community health workers, nurse from the Polo Base, nutritionist at the Polo Base, Village Leader, and parents.  Re why the university students were brought in, etc  We have amended the text, page 24  The research group at the state university of Mato Grosso do Sul has been working with indigenous communities for over a decade, and the undergraduate Indigenous students are extensively involved as junior researchers and also assist in the primary care services. The involvement of these future doctors in research allows an integration of Indigenous knowledge and worldviews to ensure that systems and services align with what is perceived to be culturally appropriate for their communities. Thus, in addition to those who took part in the study in the Terena Village, we consulted eight Indigenous undergraduate medical students on whether the findings resonated with the perspectives of their communities. This helped to strengthen the interpretation as any future intervention will need the support of these Indigenous Future Leaders. |
| 5. discussion and implications sections need to be tethered to the work of this project more specifically… more reflection on the meaning of this work … in the implications section that we learn specifics about participants and their relationships to the local school system and Polo Base. This should come earlier on in a description of the participants…. Plans/intentions..could be considered unethical to generate strong desire without a facilitated pathway | Re more reflection on the meaning  We have interspersed the meaning of the findings throughout the discussion and implications, mindful that Indigenous engagement and participation and the broader determinants of Indigenous health and well-being are often ignore in studies. Hence the emphasis on the use of appropriate methodology and making sense of the points that were raised by Indigenous young people on the broader determinants that affect their health and being. We have added a few sentences in the discussion to ensure this tethering is clear. (page 21-25)  Re implications section …specifics about participants and their relationships to the local school system and Polo Base  We hope the amendments to the methods sections addresses some of this. The section on the implications was agreed with the 8 medical students so we would prefer to leave that section as it is.  Re plans/intentions ..unethical to generate strong desire without a facilitated pathway  We are acutely aware of this point ( see response to point 1). When research funding finishes there is nervousness about how to sustain or implement findings if they are positive. The co-development ethos of this study with those who plan and provide care for young people as well as with young people themselves will hopefully help the Terena to advocate for what they felt to be important priorities. Our continued collaboration with the community has also helped to plan the next steps, which includes capacity development of young cadre of Indigenous people who can advocate for their rights. This is part of another study we have ongoing and will be reported elsewhere. |
| 6. Minor spacing issues in the text, and please note that commas and periods etc | Amended |
| 7. Some restrictions to data access will apply. This certainly makes sense given that qualitative data were collected from participants, some of whom were minors. ..not clear from the write-up what types of data would be sensitive, as images produced by the children are depicted. If some parents did not give permission .. | Supplementary file (S6_Data set) includes all anonymised statements, and cluster and point maps which were used to produce the cluster rating maps for importance and feasibility ratings. Our ethics approvals cover access to only anonymysed data. Given that concept mapping is a participatory process, all images, maps and statements included in the paper were approved by participants for publication. |
| 8 future.. all relevant parties, such as local policymakers, are included in the concept mapping process. Having adequate buy-in, funding, and support for identified solutions among stakeholders at different levels of influence… | Thank you for this important point. Policymakers were not involved in this study but they are involved in another study in progress. Here we focused on key stakeholders in the village (Village Leader, nurses, community health workers, teachers, students, parents and tribe leaders). It is our wish to ultimately bring all involved in planning and provision of care of young people to the same table with young Indigenous people to discuss these issues equal respect. |

| Reviewer 2 |  |
| --- | --- |
| 1. ..demographic information about the Indigenous population, but there is  no reference to where this information was obtained. …also information about youth suicides that does not include the source of the information. | References added – pages 26-27 |
| 2. How was the sampling purposeful? What criteria used made the sampling purposeful? | Over the decade of working with the Terena, we had mapped key stakeholders in the village and were able to therefore identify the village Leadership, primary care and education services etc For concept mapping to capture the views of those who provide and receive care it was necessary to have a broad representation from these sectors. A pragmatic decision was made to invite the Village Leader to represent the Village Tribal Council, all of the Polo Base staff (which is very small), and representation of teachers and parents of participating students in the two age groups. The total number of students in the school were 300, and 40 were invited based on the advice of teachers who took into consideration who might be present the days and/or available at the times that concept mapping sessions took place.    We have amended the methods (page 12) |
| 3. Family and Socio-economic circumstances appear to have a lot of overlap. … For example, why is statement 45, "my family" listed under Socio-economic circumstances rather than Family? | Thanks for this very important point  Statistically, the proximity of the statements points is based on which statements were more likely to have been placed in the same pile by the participants. Statements in Family and Socio-economic Circumstances clusters were very close to one another and some overlap is expected when defining the boundaries of a cluster. Regardless of the clusters, the underlying point map remains constant as it is the foundation of the MDS analysis and should be accorded more weight than the partitioning of the space in clusters.  We have added the following to the discussion (page 24).  Concept mapping illustrated the interconnectedness of issues within and across cluster boundaries, for example by the closeness of the statements on the point maps in the clusters for Family and Socio-economic Circumstances. It captured the variations in Indigenous youth perspectives, including where socio-economic circumstances were perceived to influence their family lives more so for some than others. |
