# Supplementary material for: Co-developing a health promotion programme for indigenous youths in Brazil: A concept mapping report
Source: PLoS One. 2023 Feb 15;18(2):e0269653. doi: 10.1371/journal.pone.0269653 (PMC9931109; doi:10.1371/journal.pone.0269653)
Supplement: Attachment — Submitted filename: Responses 20_12 PLOS.docx [file pone.0269653.s010.docx]

**Reviewer #1**

Reviewer #1: Great job addressing the previously expressed concerns! The following needs to be amended before publication:

Thank you
o Abstract needs editing (e.g., Latin America, not Latin American)

Thank you
o The discussion section can still be improved by adding topic sentences to each paragraph to make the point of each section clearer. Only supporting and concluding statements should follow. All else should be omitted.

We agree, we reorganized the discussion with subtopics. It gave fluency to the thoughts developed through the text.However, we believe the text is very concise, thus we did not delete paragraphs.

o There appears to be content included that pertains to an altogether different paper submission (See supplementary information COREQ PJ and letter to the editor referencing a study in the Caribbean.

Thank you. We corrected the title and footnotes.

o There are excessive and confusing supplementary files, which took hours to sort out.

We agree. We have diminished the number of supplementary files. The idea here was to provide all the data from the study.

♣ Table 2 in the supplementary files is mislabeled and should be Table 1. The text indicates it has ALL statements, but the table indicates it has examples of statements.

Thank you. We fixed the problem.

♣ Table 1 in the supplementary files is mislabeled and should be Table 2. There appears to be two of these tables in the supplementary files, but I am unsure why.

Thank you. We fixed the problem.

♣ There are two different Figure 1 and Figure 2s in the supplementary files.

Thank you. We fixed the problem.

♣ It is unclear how Figure 2 in the supplementary files is different from Go-Zone S2-P1.

Thank you. We fixed the problem.

♣ S2 Fig in the text appears to be labeled Go-Zone S2-P1 in the supplementary files.

Thank you. We fixed the problem.

♣ The reference in the text to S4 Fig does not appear to correlate with any particular Go-Zone map in the supplementary files?

Thank you. We fixed the problem.

♣ The connection between Fig 2 in the text and supplementary files is not clear.

Thank you. We fixed the problem.

♣ I do not see a S5 Fig in the supplementary files.

Thank you. We fixed the problem.

♣ I’m generally not making connections between the Go-Zone maps in the supplementary files and the text.

We explained as bellow:

Statements from each cluster are distributed in a map according to their rating for importance and feasibility. Statements from each cluster that were rated as most important and feasible are shown in the upper right-hand quadrant of the Map (Fig 2), identified as the “Go Zone”. Data obtained through sorting originates Clusters of statements that represent themes of interest and the "go zone"represents the highly rated statements. These results are complementary and, when combined, suggest agreement across participants and which themes and related statements are important for the planning of future interventions.

♣ I don’t see references to the following supplementary files in the text and believe they may be superfluous:
S1_Point_Maps; S2 Cluster Maps; and S3 Importance_CRM.

Thank you. We fixed the problem.

♣ The dataset in S6_dataset has information that is duplicative of what appears elsewhere.

Thank you. We fixed the problem.

**Response to reviewer #3**

Reviewer #3: The authors excellently responded to the requests/ requirements of the previous reviewer(s). As a first time reader/ reviewers, I was particularly struck by the value of this piece in demonstrating how to give youth voice, alongside their adult counterparts in health promotion practice.

Thank you for your comments and feedback

I noted a few things I'd like to mention, which you can adjust as the editor requests-

1) Line 135- Consider using inequitable instead of unequal as the public health fields - and others - are increasingly recognizing that equity, not equality is what we're striving for.

We agree, thank you

2) Throughout, be sure that you are consistently applying - or not- use of the Oxford comma. It seems that it is sometimes (eg: Line 419) used but not on line 147 or 180, for instance.

We have removed all Oxford comma

3) Line 203- please consider providing more description for the role of the Village Leader. I can make some assumptions about that role, but I don't know for sure who they are -- are they elected, is it honorary, are they an elder, are there multiple leaders, etc?

The leadership structure for the Terena works as: There is a group of community leaders (such as Board members), family representatives and older adults, they have the duty of electing a Village leader and a Vice-leader

4) Line 217- please add a citation, website or some additional information to point us to where we can learn about the two-decades long partnership you mention? If it doesn't exist, I believe a full concept paper on this partnership is warranted. We need more of this across many variations of university - community partnerships-- not just with indigenous people. This is a great case study, though.

We added two new references, one from the State Government Department and one from the university

5) Line 268- please provide a citation for art-enhanced concept mapping or make it more clear that you are providing the only description of that approach that exists in what follows.

We added new reference: Cooper Y, Zimmerman E. Concept Mapping: A Practical Process for Understanding and Conducting Art Education Research and Practice. Art Education. 2020;73(2):24-32.

6) Line 311- 'Go-Zone' maps-- needs a citation.

We added new reference: Trochim WM, McLinden D. Introduction to a special issue on concept mapping. Eval Program Plann. 2017;60:166-75.

7) Table 1- I would highly recommend that you use a different term than "diet". At least in the US culture, that term is increasingly being "sidelined" as it has a connotation for short-term changes to one's nutrition patterns. In this context, you are talking about one's overall nutrition pattern, it seems, so if you could use something like that- "Nutrition pattern"- instead of "diet" that would eliminated an unnecessary possible distraction. (that term appears on line 369 again).

We agree. Thank you. We changed all over the text

8) Line 434- Early in the paper (eg: abstract) as well as here, I noticed that the phrase "structural and social determinants" was used without a follow-up phrase. It is fairly obvious that you're omitting "of health," but I believe the paper would be enhanced if you would note that either throughout or the first time you mention it (eg: structural and social determinants of health (hereafter referred to as 'structural and social determinants').

We agree. Thank you. We changed all over the text

9) This is more of a philosophical issue to consider than a firm recommendation- On line 439, you all use the phrase, "The study is..." ... in fact, this is a scholarly report on an ongoing community-academic partnership. The authenticity of the relationship is palpable. I wonder if our continued use of "study" contributes to our continued patriarchal mindset in work of this sort. It is perfectly OK and good to do solid scholarly work like you present here, but I would argue that this is not a "study." It is, instead, a "scholarly report." Or just a "report." As you surely know, words matter and reinforce our ways of thinking. As we try to move away from patriarchal approaches to community engagement and public health, I hope we can shift from using language that reinforces patriarchal thinking.

We do agree and we keep consistently all over the text as report

10) Line- 531- In the implications section, it is somewhat implied with the future directions are, but I had to hunt for it. I suggest that you use a subheading "Future Directions" and complete a paragraph or two on what you plan to do next.

We do agree.

The key overarching implications that arise from the findings relate to developing processes that could allow Indigenous young people to advocate for their actionable priorities (protection of their environments, family life, education, employment etc.), capacity building with an intersectoral and participatory lens so that Indigenous communities can implement holistic health promoting strategies that integrate their cultural knowledge, and a unique valuable opportunity for strengthening Indigenous community health systems for the health of young people. Existing initiatives, such as the Health at School Program and ICHWs in each Polo Base, provide a supporting platform from which such an approach to improving community health could be developed. The Terena nurses and ICHWs from the local Polo Base were enthusiastic to co-developing a youth health programme with youths, parents, teachers and Village Leaders. ICHWs are “experience-based experts” who can be drivers for public health action and community empowerment. They have a minimum of elementary schooling, are resident in the community, understand the local geography and culture and navigate across different sectors and services to support patients in the community. Investment in a co-developed school-Polo-Base health promotion programme, with intersectoral engagement, has potential for making Indigenous health systems responsive to the inequities in youth health, to yield dividends for healthy ageing trajectories as well as for the health of the next generation
